# Supplementary material for: Oligomerization-primed coiled-coil domain interaction with Ubc13 confers processivity to TRAF6 ubiquitin ligase activity
Source: Nat Commun. 2017 Oct 9;8:814. doi: 10.1038/s41467-017-01290-0 (PMC5634496; doi:10.1038/s41467-017-01290-0)
Supplement: Supplementary file 1 — Supplementary Information [file 41467_2017_1290_MOESM1_ESM.pdf]

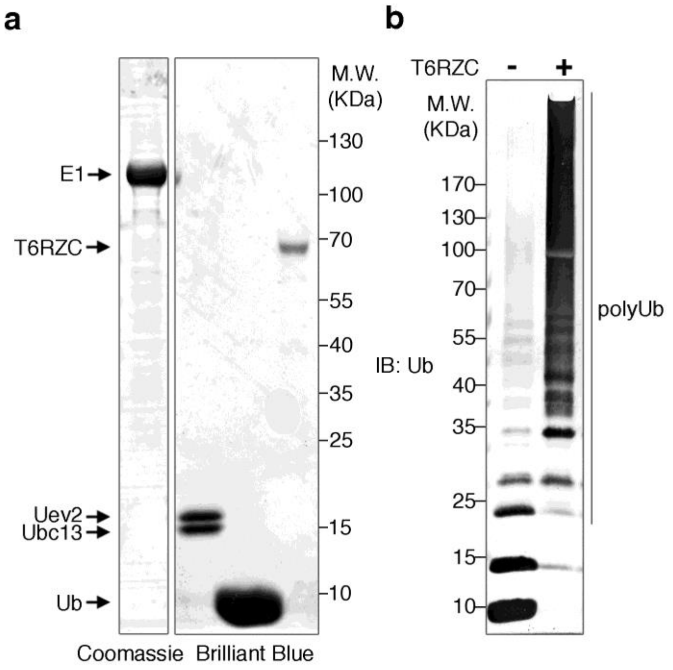


**Supplementary Figure 1.** ***In vitro*** **reconstitution of K63-linked polyubiquitin chain synthesis by TRAF6.**

(**a**) Coomassie blue staining of purified proteins. E1 (recombinant, purified from Sf9, lane 1); Ubc13/Uev2 & Ub (recombinant, purified from *E.coil*, lanes 2 and 3, respectively); T6RZC, in which the C-terminal TRAF domain was replaced with the N-terminal fragment of bacterial gyrase B (recombinant, purified from Sf9, lane 4).

(**b**) Polyubiquitin chains synthesized by TRAF6 *in vitro*. E1 (80 nM), Ubc13/Uev2 (1 μM) and ubiquitin (50 μM) were incubated with T6RZC (20 nM) for 60 min at 30 ℃. The products were resolved on 6-18% SDS-PAGE and immunoblotted with an anti-Ub antibody.

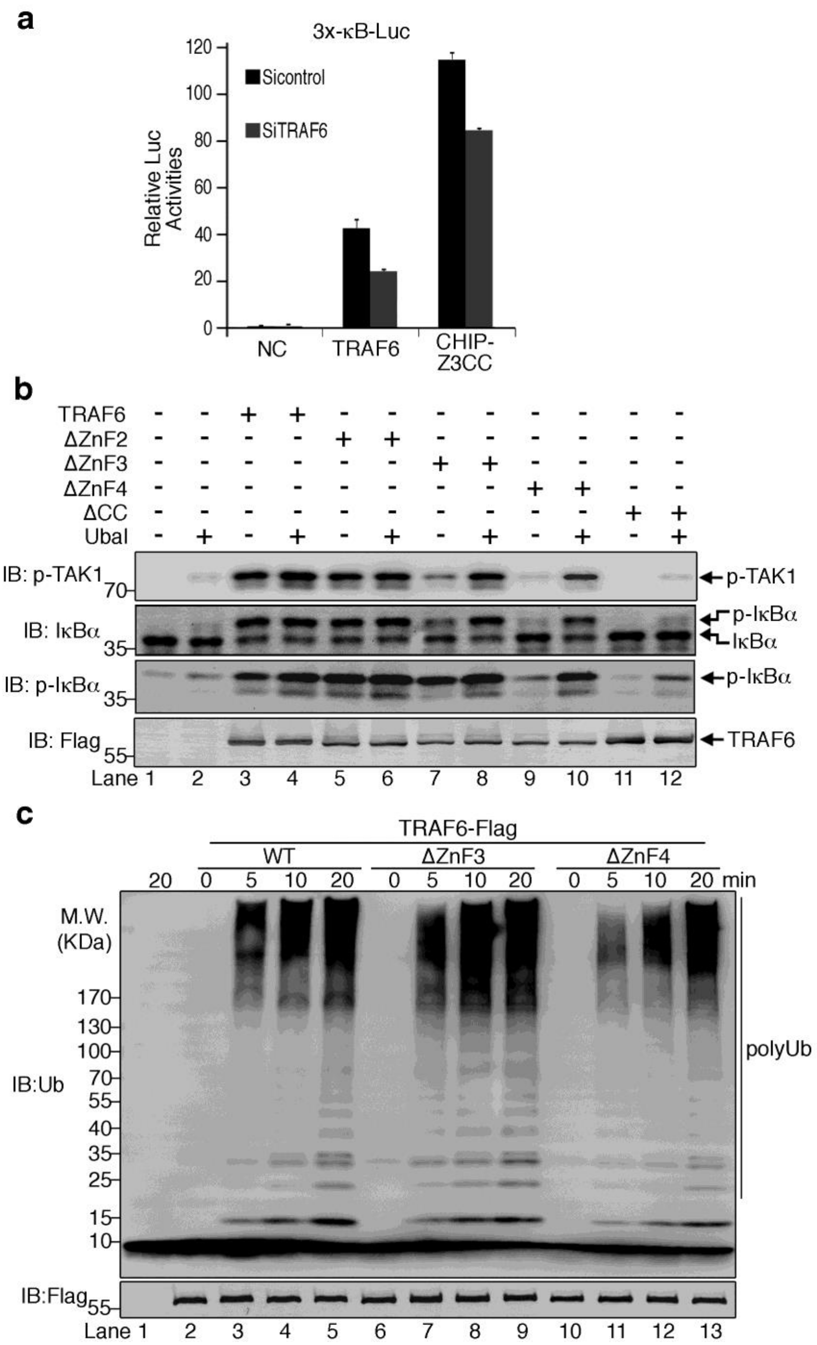


**Supplementary Figure 2. Coiled-coil domain is important for the function of TRAF6.**

(**a**) TRAF6 was not required for NF-κB activation by CHIP-Z3CC. HEK293T cells were transfected with scramble or TRAF6 siRNA oligoes. At 36 h after transfection, reporter assays were carried out with TRAF6 and CHIP-Z3CC as in Figure 2**d**. The data are showed as the mean±s.d. of triplicate independent sets of experiments.

(**b**) The activities of ΔZnF3 and ΔZnF4 mutants but not the ΔCC mutant were readily reversed by the addition of ubiquitin aldehyde (Ubal). *In vitro* IKK activation was performed with TRAF6 or the indicated deletion mutants in the absence or presence of Ubal. The products were resolved on 10% SDS-PAGE and immunoblotted with the indicated antibodies.

(**c**) TRAF6 deletion mutants ΔZnF3 and ΔZnF4 were able to synthesize comparable polyUb chains as TRAF6 WT did. As in Figure 3**c**, but the ΔZnF3 and ΔZnF4 were used.

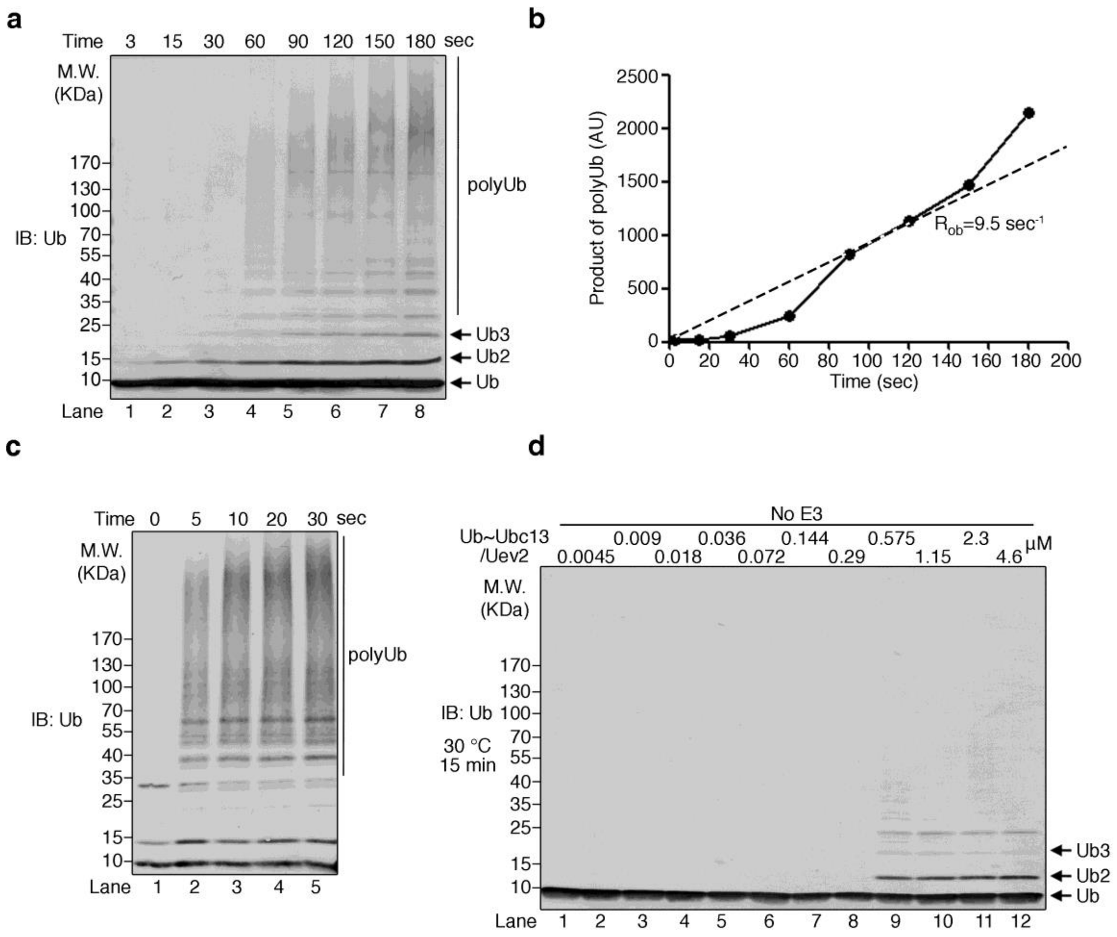


**Supplementary Figure 3. Coiled-coil domain enables processive polyUb chain synthesis by TRAF6.**

(**a**, **b**) Processive polyUb chain assembly by TRAF6. Time-course analysis of polyUb chain synthesis by T6RZC was performed as indicated and shown in (**a**). The corresponding quantification was plotted as function of time in (**b**).

(**c**) Rapid assembly of long polyUb chains. A two-step ubiquitination reaction was performed in which Ub, E1, Ubc13/Uev2 and ATP were pre-mixed for 5 minutes. Following addition of T6RZC, the reaction was incubated at 30 ℃ for the indicated duration of time. PolyUb chains were immunoblotted with an anti-Ub antibody.

(**d**) As in Figure 4**g**, but without TRAF6. The reaction products were immunoblotted with an anti-Ub antibody.

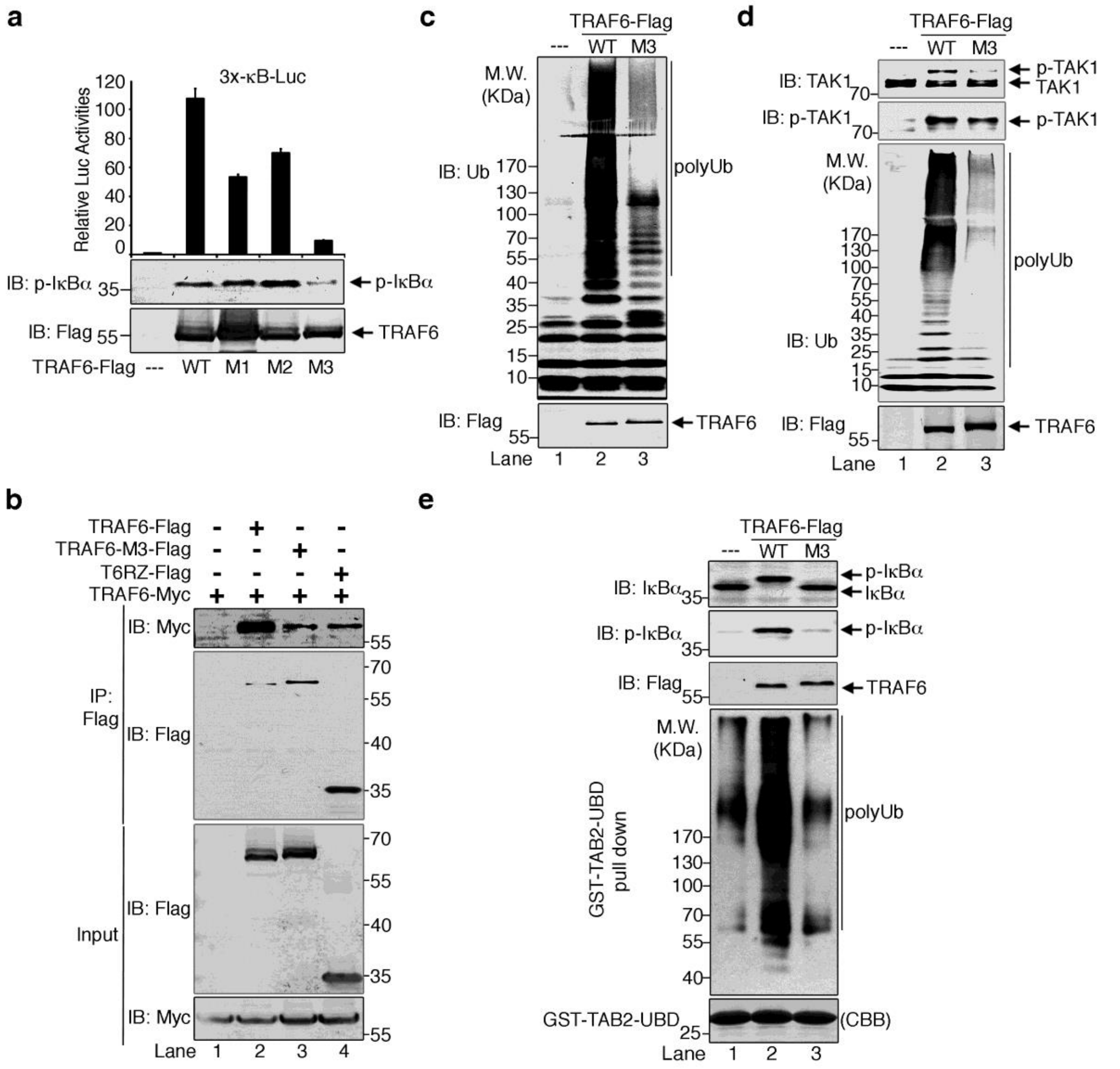


**Supplementary Figure 4. TRAF6 interaction mediated by Coiled-coil domain is important for polyUb chain synthesis and TAK1-IKK-NF-κB activation.**

(**a**) Activation of NF-κB by TRAF6 and the mutants M1, M2 and M3. As in Figure 2**d**, 3x-κB-Luc reporter assays were performed with TRAF6 and the mutants to assess their ability to activate NF-κB. The data are showed as the mean±s.d. of triplicate independent sets of experiments.

(**b**) Coiled-coil domain mediates TRAF6 self-association. Flag-tagged TRAF6, mutant M3 or T6RZ were co-transfected with Myc-tagged TRAF6 into HEK293T cells and subjected to immunoprecipitation with anti-Flag M2 beads. The immunoprecipitated complexes (upper panels) and cell lysates (down panels) were immunoblotted with the indicated antibodies.

(**c**) As in Figure 2**b**, *in vitro* ubiquitination assay was performed to measure the E3 ligase activities of TRAF6 and TRAF6-M3.

(**d**) *In vitro* TAK1 activation assay was performed to compare their TAK1 stimulatory activities between TRAF6 and TRAF6-M3.

(**e**) *In vitro* IKK activation assay was performed with TRAF6 and TRAF6-M3. In addition to the detection of IκBα and IκBα phosphorylation, the reaction products were also used to capture K63-linked polyubiquitin chains by GST-TAB2-UBD. The GST pull-down products were analyzed by immunoblotting with a K63-Ubn-specific antibody.

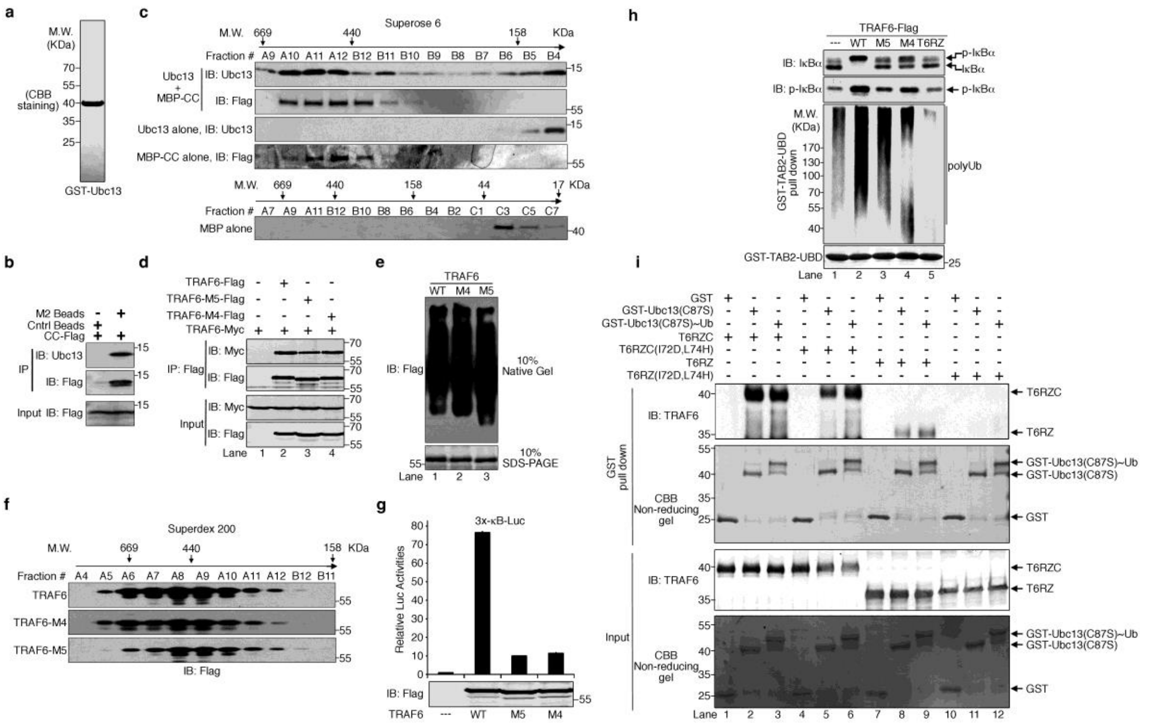


**Supplementary Figure 5. Oligomerized Coiled-coil domain interacts with Ub~Ubc13 contributing to processivity of TRAF6 E3 activity.**

(**a**) Coomassie blue staining of purified GST-Ubc13 protein.

(**b**) Coiled-coil domain interacts with Ubc13. Flag-tagged CC were overexpressed in 293T/TRAF6 KO cells and subjected to immunoprecipitation with anti-Flag M2 beads or control beads. The immunoprecipitated complexes (*upper panels*) and cell lysates (*down panels*) were immunoblotted with the indicated antibodies.

(**c**) Coiled-coil domain interacts with Ubc13. Flag- and MBP-tagged CC were expressed in 293T/TRAF6 KO cells and purified using anti-M2 beads. CC alone, Ubc13 alone, or mixture of CC and Ubc13 were subjected to size-exclusion chromatography using Superose 6. Fractions were detected using the indicated antibodies.

(**d**) TRAF6 mutants M4 and M5 don't affect TRAF6 self-association. Flag-tagged TRAF6, mutants M4 or M5 was co-transfected with Myc-tagged TRAF6 into HEK293T cells and subjected to immunoprecipitation with anti-Flag M2 beads. The immunoprecipitated complexes (*upper panels*) and cell lysates (*down panels*) were immunoblotted with the indicated antibodies.

(**e**, **f**) TRAF6 mutants M4 and M5 don't affect TRAF6 self-association. Flag-tagged TRAF6, mutants M4 or M5 were expressed in HEK 293T/TRAF6 KO cells and purified using anti-Flag M2 magnetic beads. The eluted proteins were analyzed by 10 % native gel (**e**) or by size-exclusion column Superdex200 (**f**).

(**g**) Impaired activation of NF-κB by TRAF6 mutants M4 and M5. As in Figure 2**d**, 3x-κB-Luc reporter assays were performed with TRAF6 and the mutants to assess their ability to activate NF-κB. The data are showed as the mean±s.d. of triplicate independent sets of experiments.

(**h**) Impaired *in vitro* IKK activation by TRAF6 mutants M4 and M5. *In vitro* IKK activation assay was performed with TRAF6, TRAF6-M4 and TRAF6-M5. In addition to the detection of IκBα and IκBα phosphorylation, the reaction products were also used to capture K63-linked polyubiquitin chains by GST-TAB2-UBD. The GST pull-down products were analyzed by immunoblotting with a K63-Ubn-specific antibody.

(**i**) Ub~Ubc13 interacts with RING domain and coiled-coil domain. GST-Ubc13(C87S) or Ub-loaded GST-Ubc13(C87S)~Ub was mixed with the indicated TRAF6 or mutants and subjected to GST pulldown assay. Bound proteins were separated on SDS-PAGE for TRAF6 immunoblotting or on non-reducing SDS-PAGE for Ub~Ubc13 detection using Coomassie brilliant blue (CBB) staining.

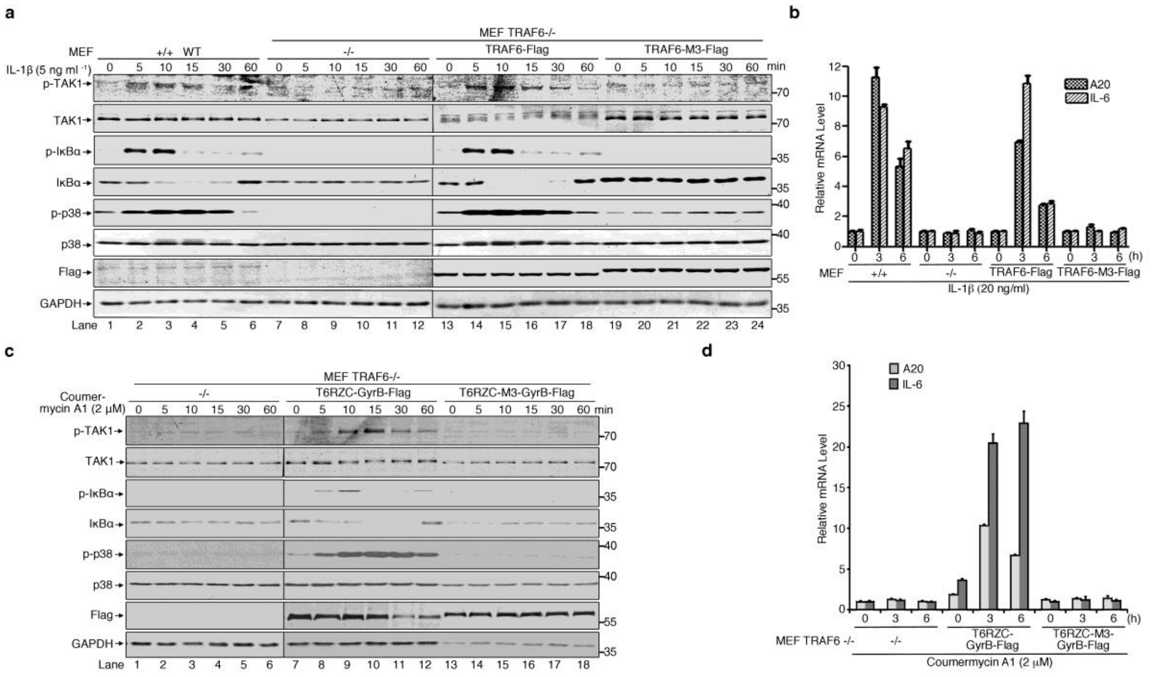


**Supplementary Figure 6. Coiled-coil domain-mediated oligomerization and interaction with Ubc13 are critical for the biological functions of TRAF6.**

(**a**, **b**) Full rescue of IL-1β signaling in TRAF6 KO MEF cells by TRAF6 wild-type (WT) but not the mutant M3. TRAF6 KO MEF cells reconstituted with TRAF6 WT or M3 mutant were stimulated with IL-1β for the indicated duration of time. Cell lysates were analyzed by immunoblotting with the indicated antibodies shown in (**a**). Total RNAs were extracted and subjected to qRT-PCR to measure the induction of IL-6 and A20 shown in (**b**), as described in experimental procedures. The data are showed as the mean±s.d. of triplicate independent sets of experiments.

(**c**, **d**) Reconstitution of NF-κB activation by T6RZC-GyrB WT but not the mutant M3. TRAF6 KO MEF cells reconstituted with Flag-tagged T6RZC-gyrase B (T6RZC-GyrB-Flag) or the mutant M3 (T6RZC-M3-GyrB-Flag) were stimulated with Coumermycin A1 for the indicated duration of time. Cell lysates were analyzed by immunoblotting with the indicated antibodies shown in (**c**). Total RNAs were extracted and subjected to qRT-PCR to measure the induction of IL-6 and A20 shown in (**d**). The data are showed as the mean±s.d. of triplicate independent sets of experiments.

Figure 1c

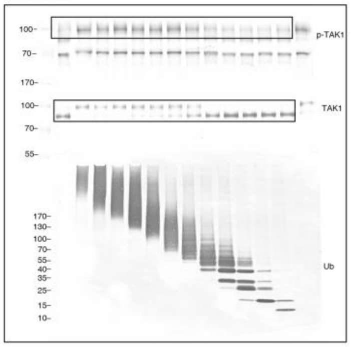


Figure 1d

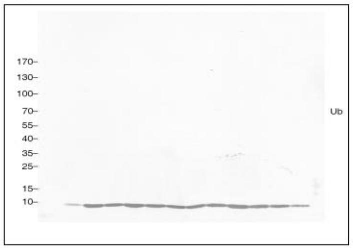


Figure 2c

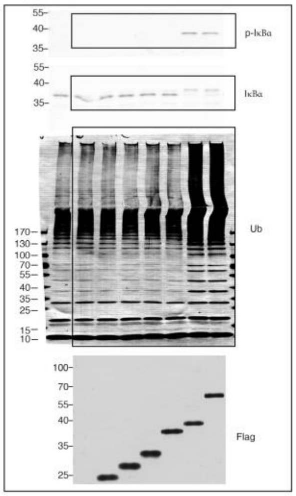


Figure 1e

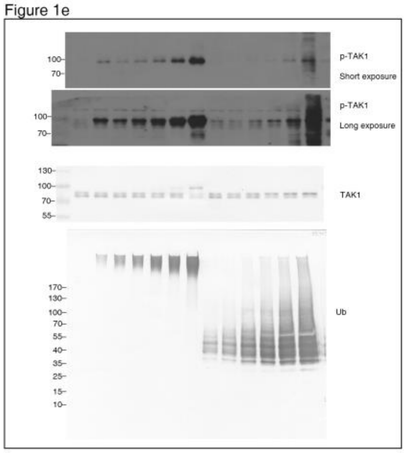


Figure 2b

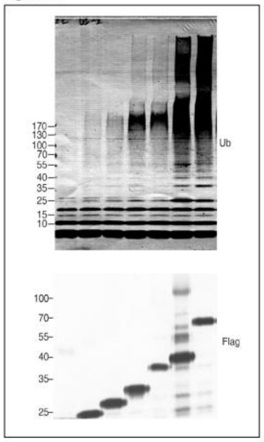


Figure 2d

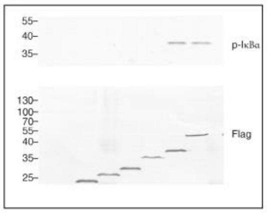


Figure 3b

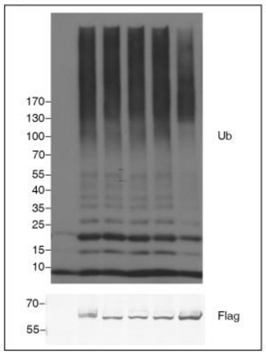


Figure 3c

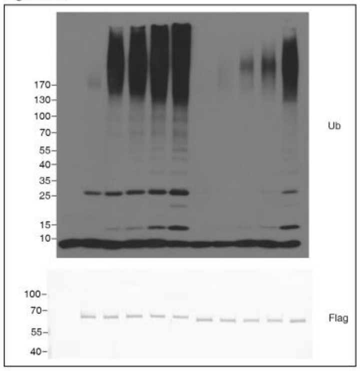


Figure 3e

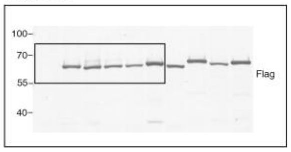

Figure 4a

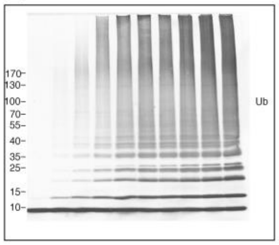


Figure 4c

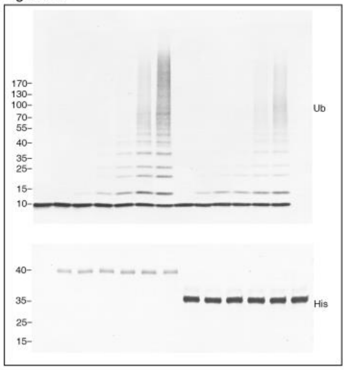


Figure 4e

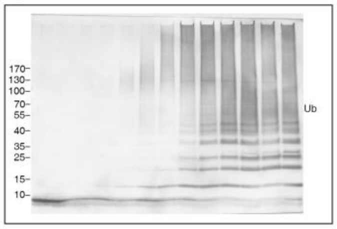


Figure 4f

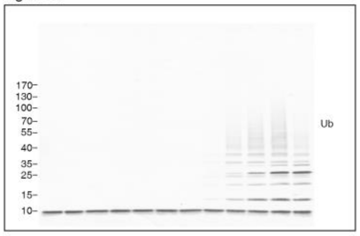


Figure 5c

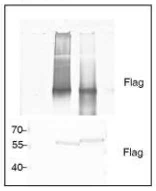


Figure 5e

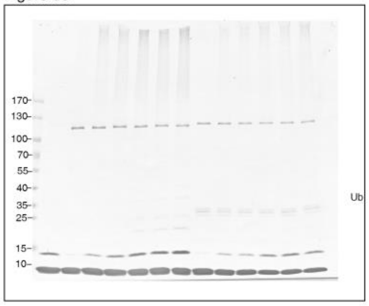


Figure 5b

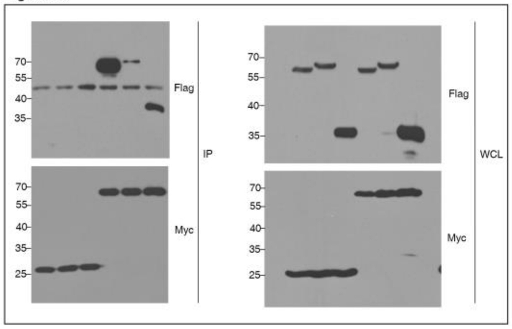


Figure 5d

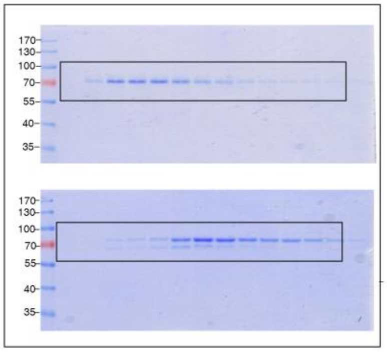

Figure 5g

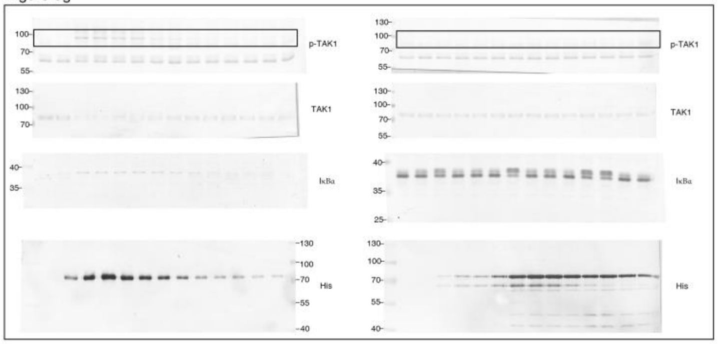

Figure 6a

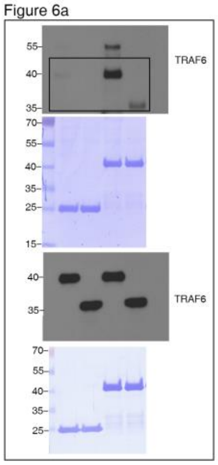


Figure 6b

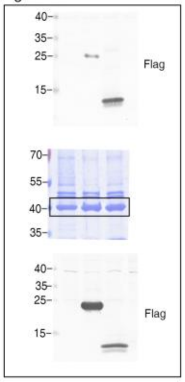


Figure 6c

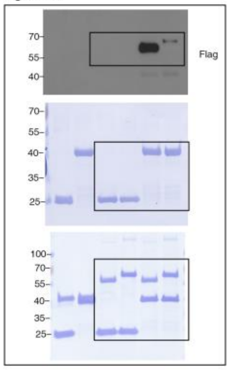


Figure 6e

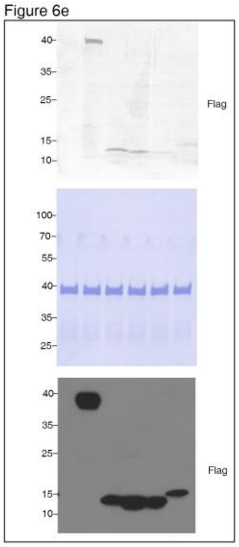


Figure 6f

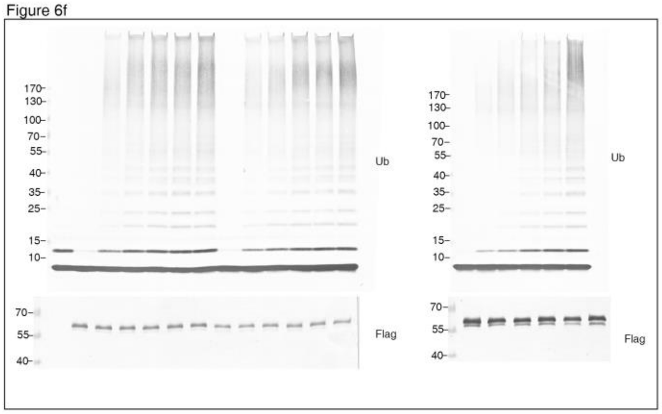


Figure 7a

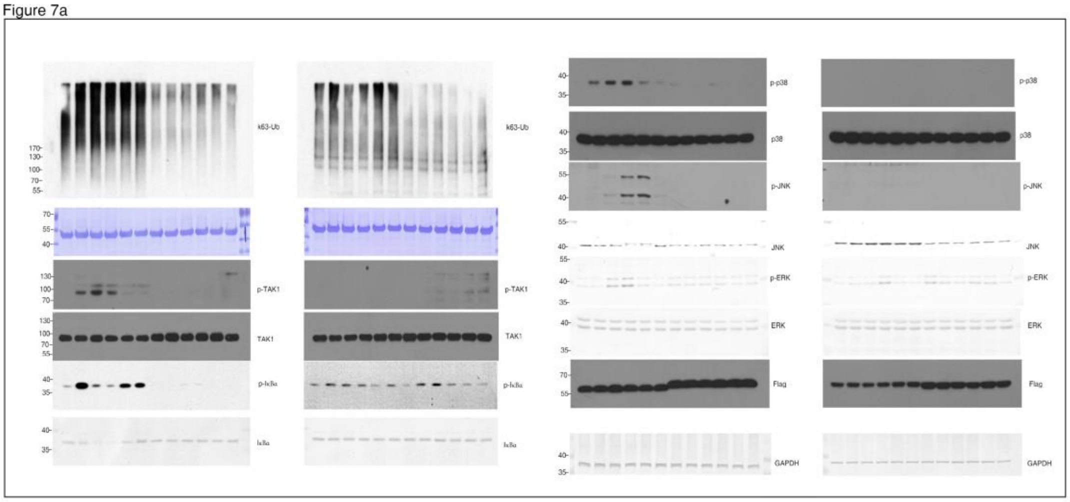

Figure S1a

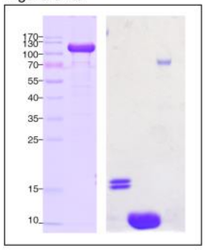


Figure S1b

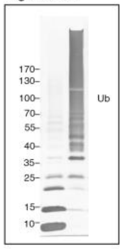


Figure S2b

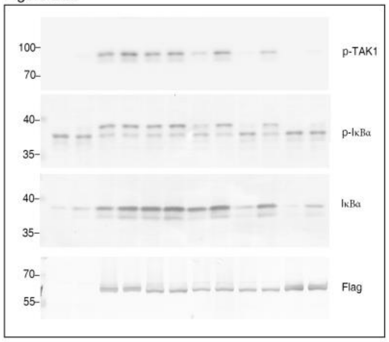


Figure S2c

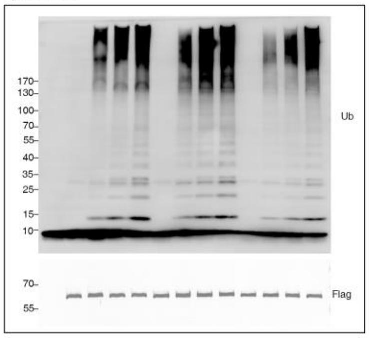


Figure S3a

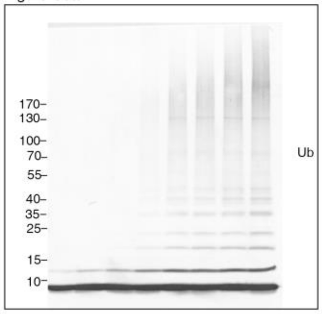


Figure S3d

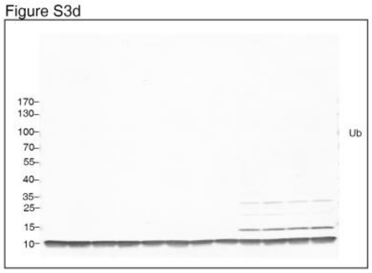


Figure S3c

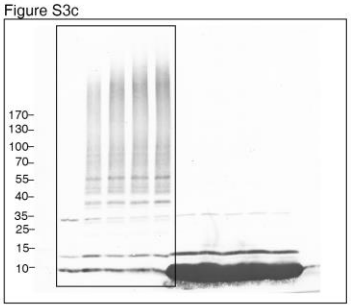


Figure S4a

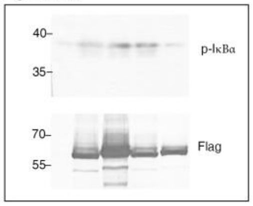


Figure S4b

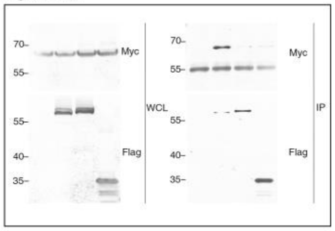


Figure S4c

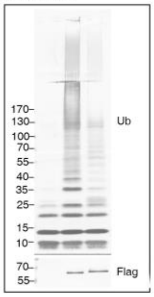


Figure S4d

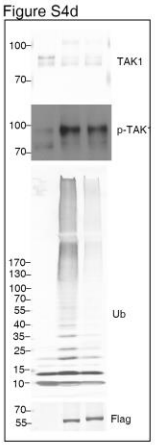


Figure S4e

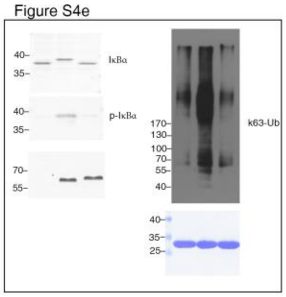

Figure S5a

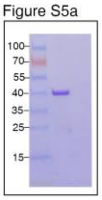


Figure S5b

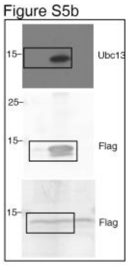


Figure S5c

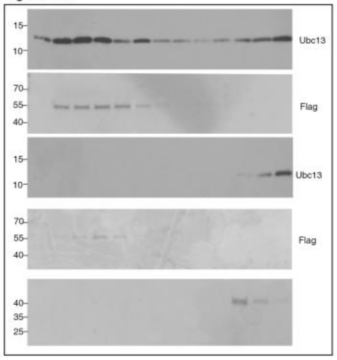


Figure S5d

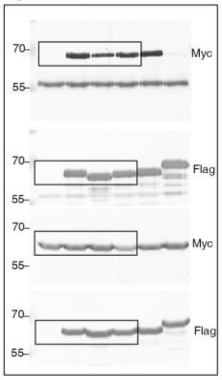


Figure S5e

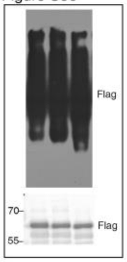


Figure S5g

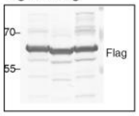


Figure S5h

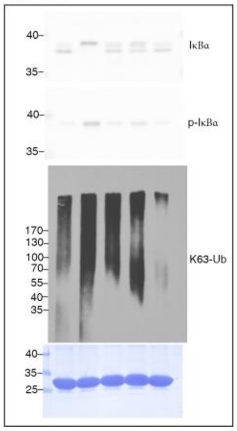


Figure S5i

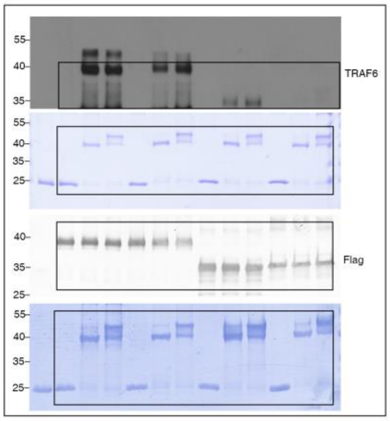


Figure S5f

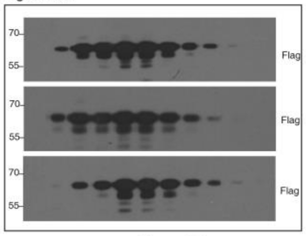


Figure S6a

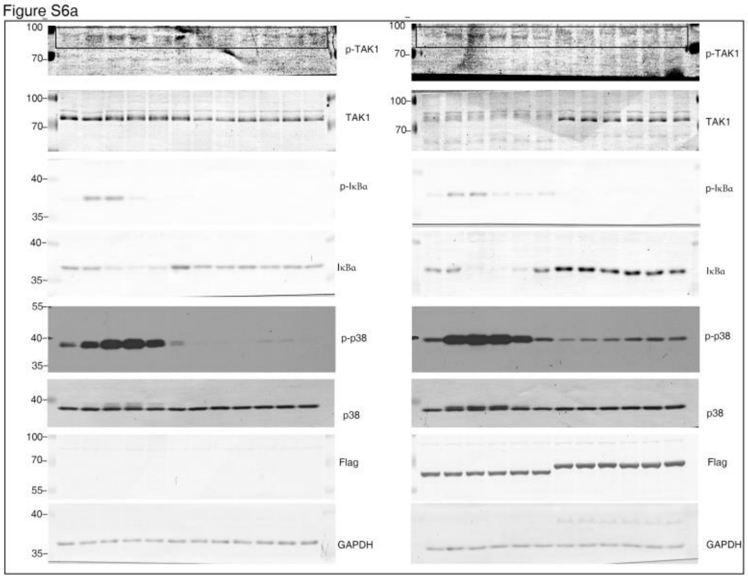

Figure S6c

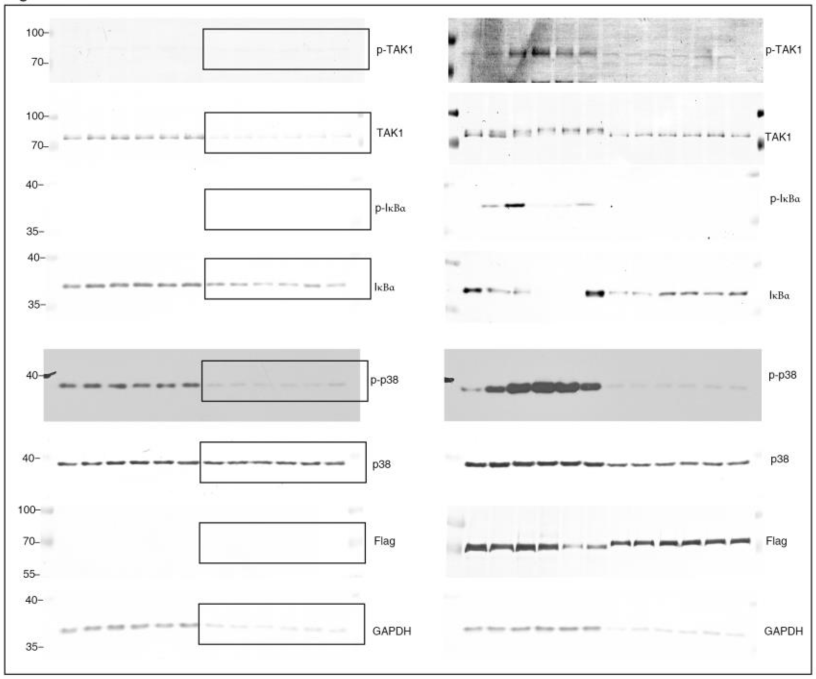


**Supplementary Figure 7. Uncropped scans of western blots included in main figures.**
